# Design, Synthesis, and Antitumor Activity Study of All-Hydrocarbon-Stapled B1-Leu Peptides

**DOI:** 10.3389/fchem.2022.840131

**Published:** 2022-04-07

**Authors:** Zhen Su, Chao Liu, Wei Cong, Shipeng He, Li Su, Honggang Hu

**Affiliations:** Institute of Translational Medicine, Shanghai University, Shanghai, China

**Keywords:** antimicrobial peptides (AMPs), cathelicidin-BF, all-hydrocarbon stapling, antitumor biological activity, B1-leu

## Abstract

B1-Leu peptide is a structural optimization compound derived from the lysine- and phenylalanine-rich antimicrobial peptide Cathelicidin-BF. It has shown promising antibacterial and antitumor biological activity. However, linear peptides are not the best choice for novel drug development due to their poor pharmacokinetic properties. In this study, various all-hydrocarbon stapled B1-Leu derivatives were designed and synthesized. Their secondary structure, protease stability, and antitumor and hemolytic activities were also investigated to evaluate their clinical value for cancer therapy. Among them, B1-L-3 and B1-L-6 showed both damaging the tumor cell membrane stability and antitumor activity, showing that they are promising lead compounds for the development of novel cancer therapeutics.

## Introduction

Antimicrobial peptides (AMPs), a class of organic endogenous protective peptides, play important roles in both biological innate immunity and acquired immunity (Tornesello et al., 2020; Hancock et al., 2021; Li et al., 2021). Ever since Boman et al. (1989) isolated cecropin from *Hyalophora cecropia* in 1975, more than a thousand species of AMPs derived from viruses, bacteria, plants, insects, mollusks, crustaceans, amphibians, fish, birds, and mammals have been confirmed and studied (Waghu et al., 2016). Research has shown that AMPs have broad-spectrum antimicrobial effects, and their sterilization mechanism makes it difficult for pathogens to produce drug-resistant mutations (Travkova et al., 2017; Savini et al., 2020). Besides, they also have excellent antitumor activity with complex mechanisms and show obvious advantages, such as a greater specificity and avoiding drug resistance, over traditional antitumor drugs (Hoskin and Ramamoorthy, 2008; Housman et al., 2014).

B1 (VKRFKKFFRKLKKSV-NH_2_) and B1-Leu peptide (B1-L, VKRFKKFFRKLKKLV-NH_2_) are both structural optimization peptides derived from lysine- and phenylalanine-rich AMP cathelicidin-BF found in the venom of the snake *Bungarus fasciatus* (Chen et al., 2011; Deng et al., 2015). Deng et al. (2015) showed that B1-Leu peptide exerts more pronounced anticancer activity than B1 peptide against drug-resistant cell lines, such as MCF-7/ADM and K562/ADM. They deduced that B1-L peptide could disrupt the cell membrane and enter the cytoplasm, then act on the mitochondria to stimulate the release of cytochrome C. For this reason, B1-Leu peptide is now considered a potential candidate for cancer therapy.

However, linear peptides may not be the best choice for novel drug development due to their poor pharmacokinetic properties. They do not readily maintain stable secondary structures and are susceptible to proteolytic degradation (Marr et al., 2006). Structure modification strategies should be used to improve their physical and chemical properties. The all-hydrocarbon peptide stapling strategy developed by Verdine and coworkers, which is realized through ring-closing metathesis between olefin-bearing amino acids, has become one of the most recognized methods of peptidomimetic development (Schafmeister et al., 2000). It can provide more rigid secondary structure and lock the desired spiral conformation, resulting in an increased protease stability, improved cell penetration, and higher target binding affinity (Bird et al., 2010; Wang and Chou, 2015; Wang et al., 2015). The aliphatic side chain can also improve the hydrophobicity of peptides, to an extent. Increased helicity, hydrophobicity, and penetrative ability are key factors in AMPs’ biological activity (Dinh et al., 2015; Luong et al., 2017; Migoń et al., 2018; Mourtada et al., 2019). For these reasons, we believe the all-hydrocarbon peptide stapling strategy could be an effective strategy for B1-Leu peptide optimization.

Herein, we describe the design and synthesis of various all-hydrocarbon stapled B1-Leu derivatives. Their secondary structures, protease stability, and antitumor and hemolytic activities were also investigated. We found that most stapled derivatives showed a higher helicity level, stronger protease stability, improved antitumor activity, and low hemolytic activity. The optimal derivatives B1-L-3 and B1-L-6 may act as promising lead compounds for the development of novel cancer therapeutics.

## Experimental Section

### General Information

Fmoc-protected amino acids were commercially available from GL Biochem (Shanghai) Ltd. Other reagents and solvents were brought from Sigma-Aldrich and Sinopharm Chemical Reagent Co., Ltd. ESI-MS was measured with a Bruker Esquire-LC mass spectrometer.

### Peptide Synthesis

Peptides were synthesized manually on Rink amide-resin (500 mg, loading capacity = 0.33 mmol/g). Normal amino acids and Fmoc-S_5_-OH and Fmoc-R_8_-OH (three equivalents) were coupled with HCTU (three equivalents) and DIPEA (nine equivalents) in DMF solution for 30 min. The Fmoc-protective group was deprotected under the treatment of 20% piperidine DMF solution. On-resin peptide precursor was subjected to first-generation Grubbs’ reagent (three equivalents) in dry dichloroethane solution for 4 h to complete olefin metathesis reaction (Wang et al., 2020). Peptide was cleavaged by B cocktail (TFA/water/phenol/TIPs = 88:5:5:2, *v/v/v/v*) for 2 h. Crude peptide was precipitated by chilling diethyl ether, and analyzed and purified by RP-HPLC. A Vydac C18 column (5 µm, 4.6 mm × 250 mm) with a 1-ml/min flow rate was used for analytical RP-HPLC, and a Vydac C18 column (10 µm, 10 mm × 250 mm or 22 mm × 150 mm) with a 3- to 6-ml/min flow rate was used for semipreparative RP-HPLC. The solvents systems were buffer A (0.1% TFA in water) and buffer B (0.1% TFA in CH_3_CN). Data were recorded and analyzed using the software system LC Solution.

B1 (VKRFKKFFRKLKKSV-NH_2_).

227 mg, 71% purified yield. ESI-MS m/z calcd. for C_94_H_160_N_28_O_16_ 1938.49; found [M + 2H]^2+^ = 970.10; [M + 3H]^3+^ = 647.20.

B1-L (VKRFKKFFRKLKKLV-NH_2_).

223 mg, 69% purified yield. ESI-MS m/z calcd. for C_97_H_166_N_28_O_15_ 1964.58; found [M + 2H]^2+^ = 983.00; [M + 3H]^3+^ = 655.95.

B1-L-1 (VS_5_RFKS_5_FFRKLKKLV-NH_2_).

209 mg, 65% purified yield. ESI-MS m/z calcd. for C_99_H_164_N_26_O_15_ 1958.57; found [M + 2H]^2+^ = 980.05; [M + 3H]^3+^ = 653.80.

B1-L-2 (VKRFS_5_KFFS_5_KLKKLV-NH_2_).

203 mg, 64% purified yield. ESI-MS m/z calcd. for C_99_H_164_N_24_O_15_ 1930.55; found [M + 2H]^2+^ = 966.20; [M + 3H]^3+^ = 644.60.

B1-L-3 (VKRFKS_5_FFRS_5_LKKLV-NH_2_).

213 mg, 66% purified yield. ESI-MS m/z calcd. for C_99_H_164_N_26_O_15_ 1958.57; found [M + 2H]^2+^ = 980.25; [M + 3H]^3+^ = 653.85.

B1-L-4 (VKRFKKFFS_5_KLKS_5_LV-NH_2_).

216 mg, 68% purified yield. ESI-MS m/z calcd. for C_99_H_164_N_24_O_15_ 1930.55; found [M + 2H]^2+^ = 966.20; [M + 3H]^3+^ = 644.60.

B1-L-5 (VR_8_RFKKFFS_5_KLKKLV-NH_2_).

201 mg, 62% purified yield. ESI-MS m/z calcd. for C_102_H_170_N_24_O_15_ 1972.64; found [M + 2H]^2+^ = 987.45; [M + 3H]^3+^ = 658.50.

B1-L-6 (VKRFR_8_KFFRKLS_5_KLV-NH_2_).

214 mg, 65% purified yield. ESI-MS m/z calcd. for C_102_H_170_N_26_O_15_ 2000.65; found [M + 2H]^2+^ = 1001.50; [M + 3H]^3+^ = 667.95.

B1-L-7 (VKRFKR_8_FFRKLKS_5_LV-NH_2_).

204 mg, 62% purified yield. ESI-MS m/z calcd. for C_102_H_170_N_26_O_15_ 2000.65; found [M + 2H]^2+^ = 1001.30; [M + 3H]^3+^ = 667.90.

### Protease Stability Experiment

Peptides in DMSO solution (1 mM) was mixed with α-chymotrypsin in PBS buffer (0.5 ng/μl) containing 2 mM CaCl_2_ and incubated at 37°C. The percentage of peptide was measured by HPLC at 0, 5, 10, 20, 30, and 60 min(Wang et al., 2020).

### Circular Dichroism Spectroscopy Study

Peptides were dissolved in TFE/water (1:1, *v/v*, 0.1 mg/ml). CD data were recorded in 10-mm path length, a quartz cell at 20°C. Percent helicity was calculated by the following equation (Wang et al., 2006).

$$\alpha =\frac{{\left[\theta \right]}_{222}}{{\left[\theta \right]}_{\max}}\times 100\%$$

[θ]_222_ is the molar ellipticity of 222 nm; [θ]_max_ = (−44,000 + 250T) (1 − k/n), where k = 4, n is the numbers of amino acids, and T = 20°C.

### Cell Culture and Cell Viability Assay

MCF-7, HUH-7, U87, and Nthy-ori 3-1 cell lines were used to test cell viability. All the cell lines were cultured in a humid environment at 37°C with 5% CO_2_. The anticancer activity of B1 and its derivatives were evaluated by the CCK-8 reagent. All peptides tested were dissolved in DMSO and finally added to each well in 1‰ volume. The cells were seeded in a 96-well plate at a number of 5 × 10^3^ cells per well. After overnight incubation, they were administered with different concentrations of peptides. Ten percent CCK-8 solution without FBS was added to each well after 48 h. After incubation at 37°C for 30 min, the optical density (OD) at 450 nm was recorded with a microplate reader (Deng et al., 2015).

### Scanning Electron Microscopy

MCF-7 cells were seeded into a six-well microtiter plate containing sterile coverslips at a rate of no less than 1 × 10^5^ cells per well. After 24 h of incubation, 50 μM B1, 12.5 μM B1-Leu, and 5 μM B1-Leu-3 were added. After 30 min of incubation, the cells were fixed with 2.5% glutaraldehyde solution and observed by a scanning electron microscope (Deng et al., 2015).

### Hemolysis Test

Two hundred microliters of 4% rabbit red blood cells in a 96-well plate was added different concentrations of peptides. After 1 h of incubation at 37°C and following centrifugation, the release of hemoglobin was monitored by the absorbance at 450 nm of supernatant. Zero hemolysis was determined without adding any other reagents, and 100% hemolysis was determined in 10% Triton X-100. Hemolysis percentage hemolysis was calculated as: (A_peptide_ − A_blank_)/(A_Triton_ − A_blank_) × 100%. All hemolysis determinations were conducted in three repeated experiments (Deng et al., 2015).

### Fluorescence-Activated Cell Sorting

Take HUH-7 cell line, respectively, in a 24-well plate, and incubate with no less than 1 × 10^5^ cells in each well overnight. After 24 h of treatment with different concentrations of B1-L, B1-L-3, and B1-L-6, cell apoptosis was detected with annexin V-FITC/PI staining, and flow cytometry was used to analyze cell apoptosis (Li et al., 2017).

## Results and Discussion

### Design and Synthesis of Stapled B1-Leu Peptides

**SCHEME 1:**
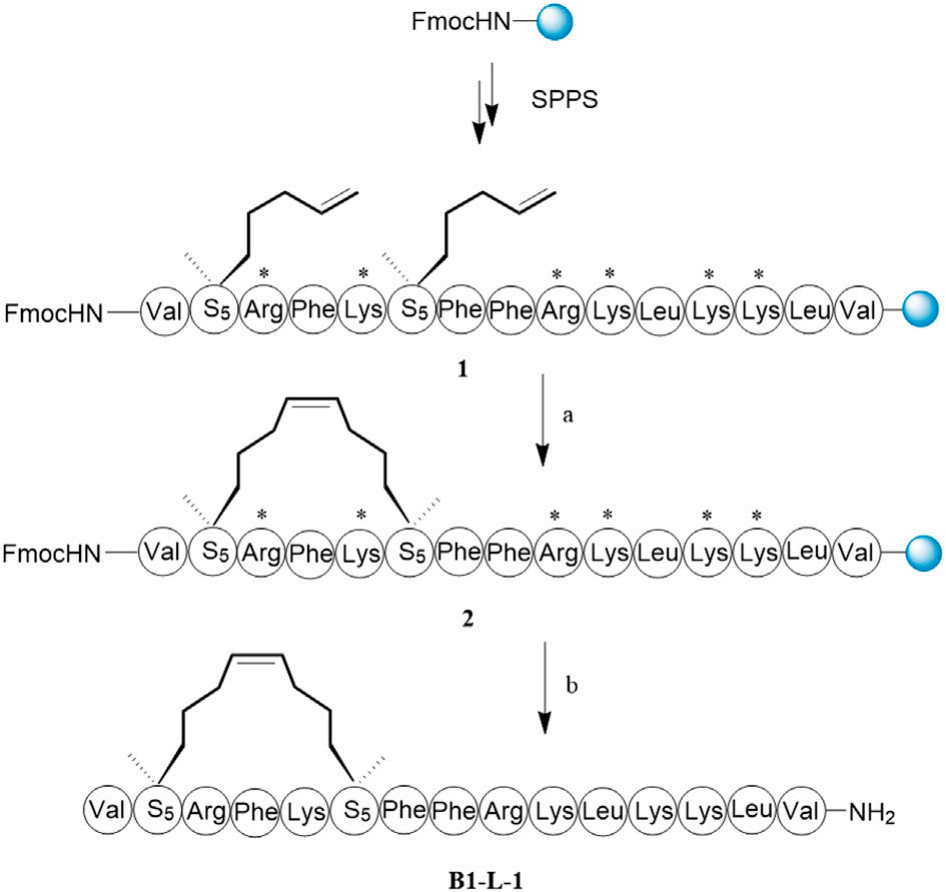
The synthesis route of B1-L-1. Reagents and conditions: **(A)** First-generation Grubbs’ reagent, DCE, 4 h, rt. **(B)** i) 20% piperdine/DMF, 20 min, rt; ii) TFA/water/phenol/TIPs = 88:5:5:2, *v/v/v/v*, 2 h, rt, 65%.

It was important that key residues significant for biological activity should be well preserved when we design all-hydrocarbon-stapled B1-Leu peptides. According to a previous study, amino acid residues with hydrophobicity or positive charges play pivotal roles in the activity of B1-Leu peptide (Deng et al., 2015). Therefore, phenylalanine, leucine, and at least one arginine were left intact. Other residues were substituted with (S)-2-(4-pentenyl) alanine amino acid (S_5_) and (R)-2-(7-octenyl) alanine amino acid (R_8_), according to the i + 4 and i + 7 strategy, to produce a series of stapled B1-Leu peptides (B1-L-1-7, Table 1) (Kutchukian et al., 2009). The following stapled peptide syntheses began with Rink amide AM resin as solid support. Taking B1-L-1 as an example (Scheme 1), HCTU and DIPEA were used as the coupling reagent to elongate the peptide backbone. The intramolecular olefin metathesis reaction of on-resin peptide 1 by the first-generation Grubbs’ reagent afforded on-resin stapled peptide 2. After the deprotection of the Fmoc group, peptide cleavage and side chain deprotection using reagent B yielded the crude B1-L-1. Further purification by RP-HPLC produced target product with over 95% purity and 65% total yield. Finally, molecular weights were confirmed by ESI-MS, which matched to the theoretical ones.

**TABLE 1:** Sequences, α-helicity, degradation half-life, hemolysis, and antitumor activity of B1, B1-Leu, and its stapled derivatives.

Peptide	Sequence	Helicity (%)	t_1/2_ (min)^a^	HC_50_ (μM)^b^	IC_50_ (μM)^c^		
					HUH-7	MCF-7	U87
B1	VKRFKKFFRKLKKSV-NH_2_	8.9	8.3	>80	>50	>50	>50
B1-Leu	VKRFKKFFRKLKKLV-NH_2_	23.5	8.5	>80	11.81 ± 0.72	13.43 ± 0.53	16.19 ± 3.32
B1-L-1	VS_5_RFKS_5_FFRKLKKLV-NH_2_	47.2	>60	>80	13.87 ± 1.25	15.89 ± 0.92	12.24 ± 1.23
B1-L-2	VKRFS_5_KFFS_5_KLKKLV-NH_2_	35.5	>60	>80	6.18 ± 0.63	6.591 ± 0.42	8.86 ± 1.43
B1-L-3	VKRFKS_5_FFRS_5_LKKLV-NH_2_	37	>60	>80	5.96 ± 0.54	5.478 ± 0.89	3.59 ± 1.20
B1-L-4	VKRFKKFFS_5_KLKS_5_LV-NH_2_	35	>60	>80	13.41 ± 1.92	15.20 ± 1.02	17.91 ± 3.23
B1-L-5	VR_8_RFKKFFS_5_KLKKLV-NH_2_	24.8	>60	>80	13.84 ± 1.53	13.99 ± 0.73	14.33 ± 2.35
B1-L-6	VKRFR_8_KFFRKLS_5_KLV-NH_2_	39.8	55.3	>80	5.89 ± 0.43	5.152 ± 1.04	4.58 ± 0.93
B1-L-7	VKRFKR_8_FFRKLKS_5_LV-NH_2_	31.6	>60	>80	7.49 ± 1.2	13.36 ± 1.22	12.70 ± 1.63

Note:

a Hydrolysis enzyme degradation half-life.

b Half hemolysis concentration.

c Half maximal inhibitory concentration.

### Degree of Helicity and Protease Stability Analysis

**FIGURE 1:**
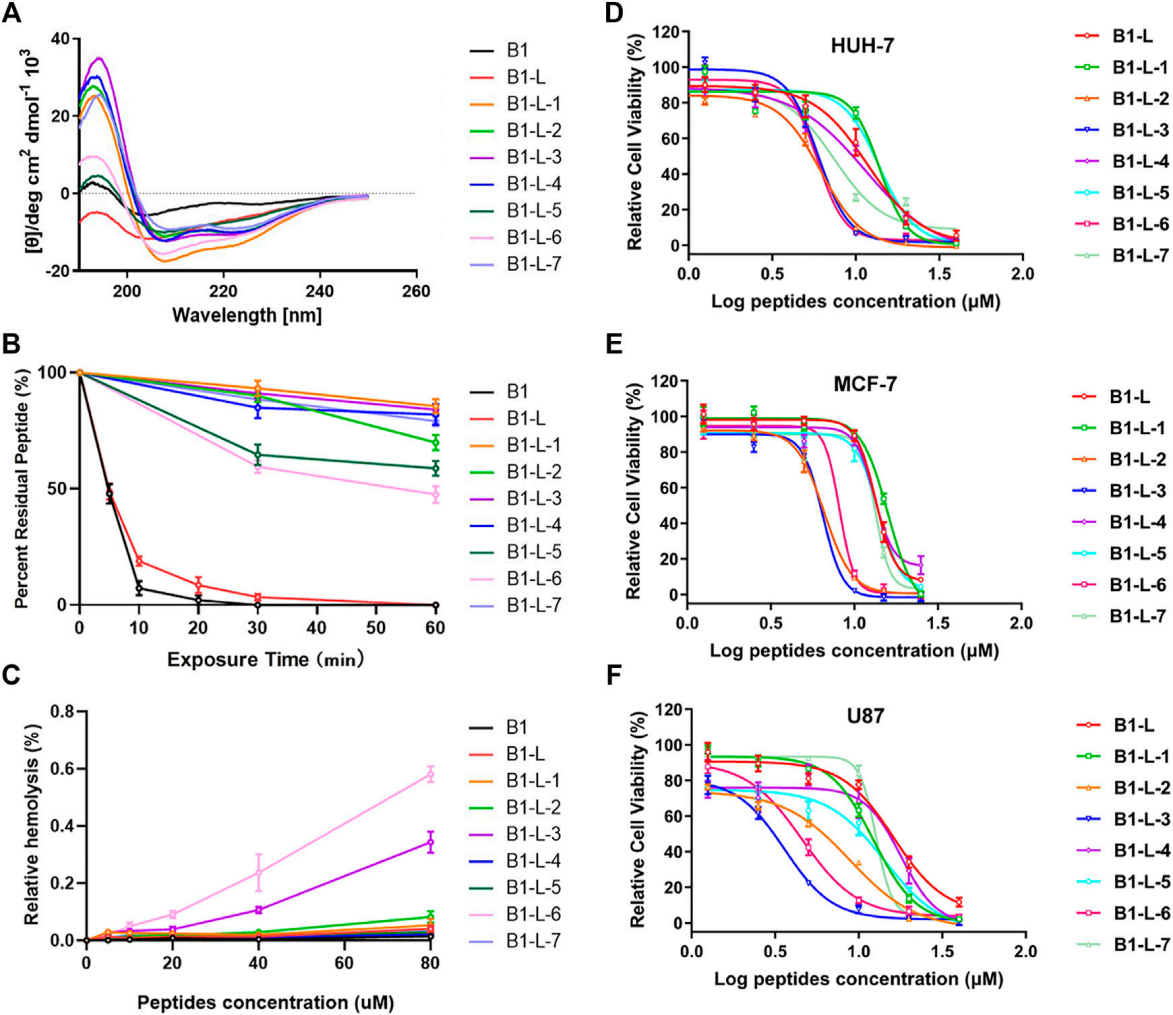
The circular dichroism data **(A)**, proteolytic stability **(B)**, hemolytic activity **(C)** and antitumor activity in different cell lines **(D–F)** of B1, B1-Leu, and their stapled derivatives. Data points were displayed as the mean value SEM of three repeated independent experiments **(B–F)**.

The secondary structures of stapled B1-Leu peptides were measured using circular dichroism (CD). CD analysis indicated that the helicities of linear peptide B1 and B1-Leu were 8.9% and 23.5%, respectively, while all the stapled peptides exhibited an improved helicity level ranging from 24.8% to 47.2% (Figure 1A and Table 1). These results showed that the all-hydrocarbon stapling strategy indeed improved helicity compared with their linear counterpart. Among them, B1-L-1, B1-L-3, and B1-L-6 showed the three highest levels of helicity (47.2%, 37%, and 39.8%, respectively). Furthermore, the α-chymotrypsin-mediated protease stability experiment showed that B1 and B1-Leu were completely degraded during 1 h of protease exposure. Meanwhile, at least 45% of stapled peptide remained intact, suggesting excellent protease stability over the linear prototype peptide (Figure 1B and Table 1). Notably, B1-L-6 showed a high-level of helicity but the weakest protease stability among these stapled peptides, indicating that secondary structure is not the only determining factor for protease stability.

### AntiTumor Activity

Next, their antitumor activities were measured in different cell lines (HUH-7, MCF-7, and U87) using the CCK-8 test (Figures 1D–F). As summarized in Table 1, B1-L-2, B1-L-3, and B1-L-6 exhibited more activity than the prototype peptides B1 and B1-Leu. Taking CD study and α-chymotrypsin experiments into account, all-hydrocarbon stapling strategy obviously improved protease stability and secondary structure, which are both significant to the antitumor activity of B1-Leu and its analogs. All-hydrocarbon side chains, which provide improved hydrophobicity, are another cause of the increased activity. However, other stapling peptides showed relatively high levels of both secondary structure and protease stability, but they failed to show better antitumor activity. One plausible explanation is that the level of helicity and protease stability were important, but were not the sole determinants of the antitumor activity of B1-Leu peptide derivatives, indicating that there may be other mechanisms, such as their ability to selectively interact with ion channels (Gkeka and Sarkisov, 2010). Finally, the cytotoxicity of B1-Leu, B1-L-3, and B1-L-6 was evaluated on Nthy-ori 3-1 cell line. As shown in Supplementary Figure S10, the IC_50_ of B1-Leu, B1-L-3, and B1-L-6 was similar to that of tumor cells in normal cells, indicating that they were not selective for tumor cells, such as most antitumor drugs.

### Membrane Disruption Effect

**FIGURE 2:**
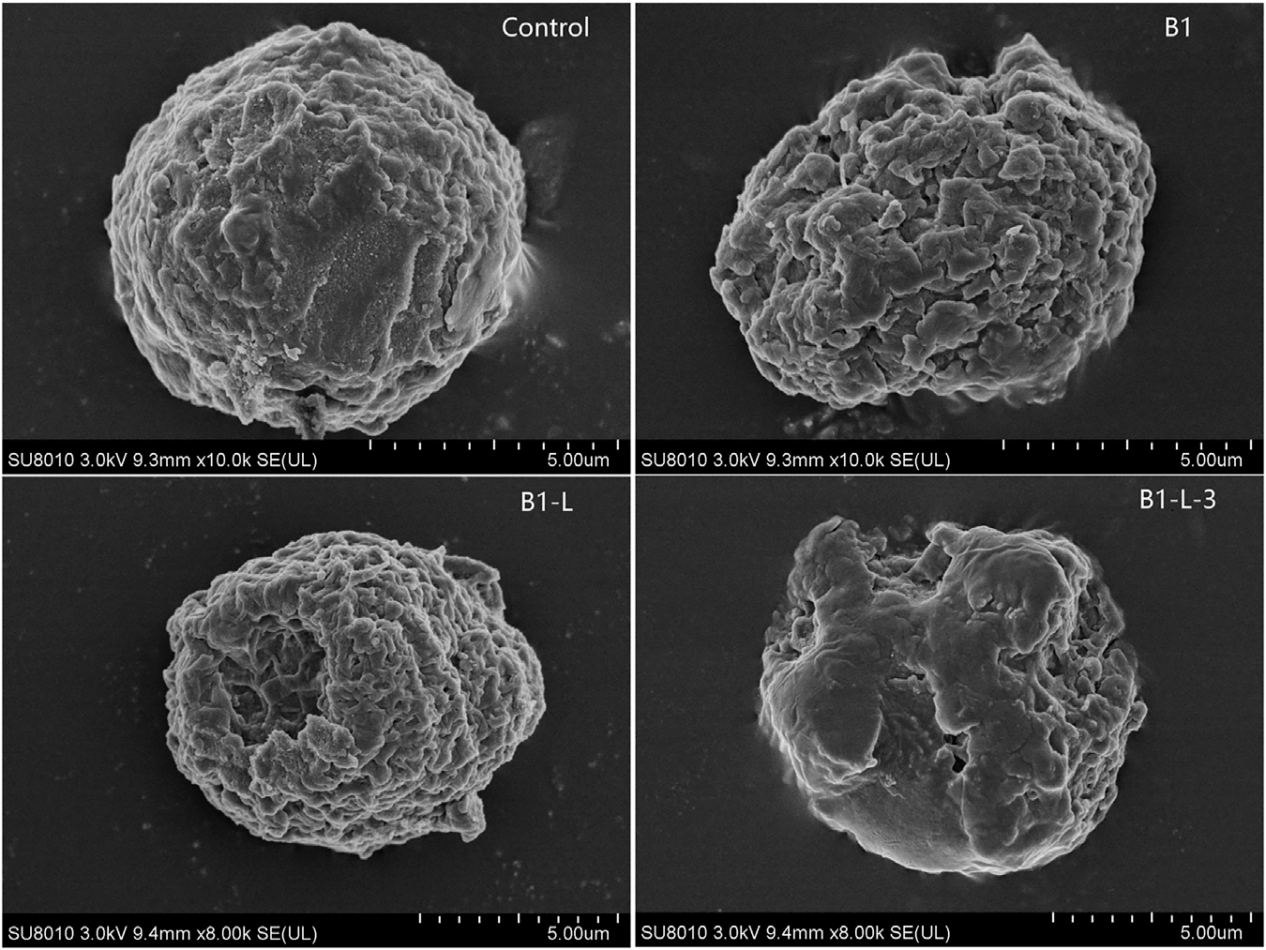
Peptides induced variations in membrane morphology of MCF-7 cell detected by scanning electron microscopy (SEM).

Membrane disruption study was visualized by SEM in MCF-7 cells. As shown in Figure 2, after incubation with prototype peptides B1, B1-Leu, and stapled peptide B1-L-3, significant variations in membrane morphology were detected in the B1-L-3 group over the others. According to the previous report (Deng et al., 2015), B1 and its analogs acted on the cell membrane and altered the permeability of cancer cells to facilitate their entry into the cells. This phenomenon was more pronounced on the staple derivative.

### Hemolytic Activity

Further hemolytic activity research was evaluated by assessing the release of hemoglobin from erythrocyte suspensions of fresh rabbit blood. As shown in Figure 1C, prototype peptides and stapled derivatives did not show significant hemolytic activity (<0.1%) at IC_50_. Even when the concentration was increased to 80 μM, the hemolytic activity of the stapled peptides still remained low (<0.6%). In this way, the all-hydrocarbon stapling strategy did not have a significant influence on the hemolytic activity of the prototype peptide.

### Induction of Cell Apoptosis

**FIGURE 3:**
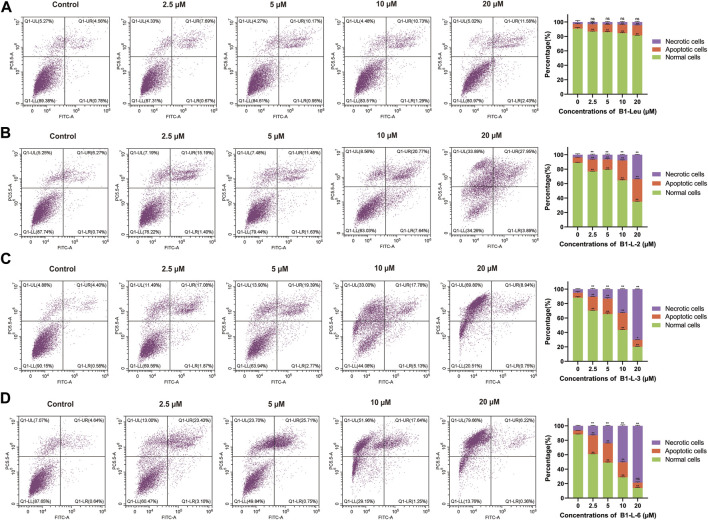
Induced apoptosis of Huh-7 cells based on staple peptides. Apoptosis of B1-Leu **(A)**, B1-L-2 **(B)**, B1-L-3 **(C)** and B1-L-6 **(D)** treated cells was analyzed by flow cytometry with annexin V-FITC/PI staining. (Representative flow cytometry plots of three independent experiments are shown, and the statistics on the right represent the mean of ± SE for three independent experiments. *p < 0.05, **p < 0.01, ns indicates no statistical difference).

The induction of apoptosis was evaluated by fluorescence-activated cell sorting with the B1-Leu peptide and most active stapled peptides B1-L-2, B1-L-3, and B1-L-6 on HUH-7 cells (Figure 3). When the peptide concentrations increased from 0 to 20 μM, different degrees of cell apoptosis and necrosis were observed. The most pronounced difference appeared at 10 μM. The percentages of apoptotic cells for B1-L-2, B1-L-3, and B1-L-6 were 16.36%, 29.03%, and 26.68%, respectively, and the percentage of cells with B1-Leu was 6.78%. When the concentration was increased to 20 μM, considerable cell necrosis was observed for B1-L-3 (90.38%) and B1-L-6 (96.84%). These results indicated that B1-Leu peptide and their stapled derivatives caused a dose-dependent increase in their apoptotic effect, and that stapled peptides exerted stronger apoptosis-inducing effects than the linear prototype peptide.

## Conclusion

In conclusion, a novel series of stapled B1-Leu derivatives was designed and successfully synthesized. The CD and protease stability study suggested that the all-hydrocarbon strategy indeed markedly improved the helicity and protease stability of the prototype linear peptide. *In vitro* antitumor inhibition tests showed that most analogs had more antitumor activity than the original peptide. However, B1-L-4 showed relatively high helicity and protease stability but poor antitumor activity, indicating that the antitumor activity of B1-Leu analogs was not only determined by their ability to damage the cell membrane but may also have been related to some other mechanisms. Subsequent cell flow cytometry research and electron microscopy showed that stapled peptides could induce stronger cell apoptosis effects than linear peptide, and that damaging the tumor cell membrane was one of their antitumor mechanisms. Finally, the hemolytic activity of all the prototype peptides and stapled derivatives remained low, suggesting that the all-hydrocarbon stapling strategy did not exert significant influence on hemolytic activity. The all-hydrocarbon stapling strategy in this work effectively ameliorated the helicity level, protease stability, and antitumor activity of B1-Leu peptide, providing a successful example of AMP structure optimization. More importantly, B1-L-3 and B1-L-6 showed both improved stability and antitumor activity, establishing them as promising lead compounds for the development of novel cancer therapeutics.

## Data Availability

The original contributions presented in the study are included in the article/Supplementary Material, further inquiries can be directed to the corresponding authors.
