# Corrigendum: Design, synthesis, and antitumor activity study of all-hydrocarbon-stapled B1-Leu peptides

**DOI:** 10.3389/fchem.2023.1285116

**Published:** 2023-10-27

**Authors:** Zhen Su, Chao Liu, Wei Cong, Shipeng He, Li Su, Honggang Hu

**Affiliations:** Institute of Translational Medicine, Shanghai University, Shanghai, China

**Keywords:** antimicrobial peptides (AMPs), cathelicidin-BF, all-hydrocarbon stapling, antitumor biological activity, B1-Leu

In the published article, there was an error in Figure 3. The figure lacked statistical results in the efficacy evaluation section. We repeated the experiment three times and counted the results. The experimental data in the figure are basically consistent with the apoptosis trend of the original figure, and the interpretation of the results remains unchanged. The corrected Figure 3 and its caption appear below.

**FIGURE 3:**
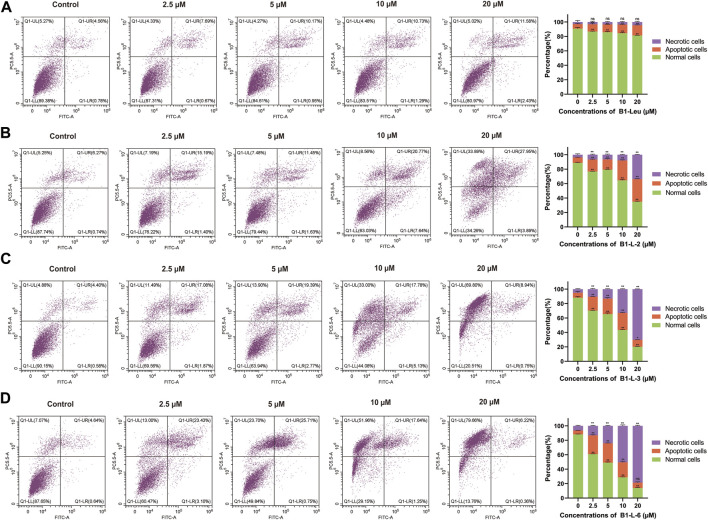
Induced apoptosis of Huh-7 cells based on staple peptides. Apoptosis of B1-Leu **(A)**, B1-L-2 **(B)**, B1-L-3 **(C)** and B1-L-6 **(D)** treated cells was analyzed by flow cytometry with annexin V-FITC/PI staining. (Representative flow cytometry plots of three independent experiments are shown, and the statistics on the right represent the mean of ± SE for three independent experiments. *p < 0.05, **p < 0.01, ns indicates no statistical difference).

The authors apologize for this error and state that this does not change the scientific conclusions of the article in any way. The original article has been updated.

