# Supplementary material for: Design, Synthesis, and Antitumor Activity Study of All-Hydrocarbon-Stapled B1-Leu Peptides
Source: Front Chem. 2022 Apr 7;10:840131. doi: 10.3389/fchem.2022.840131 (PMC9021566; doi:10.3389/fchem.2022.840131)
Supplement: Supplementary file 1 [file DataSheet1.docx]

Supplementary Material

**HPLC and HRMS spectra of compounds**

**Supplementary Figure 1.** A). The HPLC of purified B1; B) ESI-MS spectrum of B1 calcd. for C_94_H_160_N_28_O_16_ 1938.49; found [M+2H]^2+^=970.10; [M+3H]^3+^= 647.20.

**Supplementary Figure 2.** A). The HPLC of purified B1-L; B) ESI-MS spectrum of B1-L calcd. for C_97_H_166_N_28_O_15_ 1964.58; found [M+2H]^2+^=983.00; [M+3H]^3+^=655.95.

**Supplementary Figure 3.** A). The HPLC of purified B1-L-1; B) ESI-MS spectrum of B1-L-1 calcd. for C_99_H_164_N_26_O_15_ 1958.57; found [M+2H]^2+^=980.05; [M+3H]^3+^= 653.80.

**Supplementary Figure 4.** A). The HPLC of purified B1-L-2; B) ESI-MS spectrum of B1-L-2 calcd. for C_99_H_164_N_24_O_15_ 1930.55; found [M+2H]^2+^=966.20; [M+3H]^3+^= 644.60.

**Supplementary Figure 5.** A). The HPLC of purified B1-L-3; B) ESI-MS spectrum of B1-L-3 calcd. for C_99_H_164_N_26_O_15_ 1958.57; found [M+2H]^2+^=980.25; [M+3H]^3+^= 653.85.

**Supplementary Figure 6.** A). The HPLC of purified B1-L-4; B) ESI-MS spectrum of B1-L-4 calcd. for C_99_H_164_N_24_O_15_ 1930.55; found [M+2H]^2+^=966.15; [M+3H]^3+^=644.55.

**Supplementary Figure 7.** A). The HPLC of purified B1-L-5; B) ESI-MS spectrum of B1-L-5 calcd. for C_102_H_170_N_24_O_15_ 1972.64; found [M+2H]^2+^=987.45; [M+3H]^3+^= 658.50.

**Supplementary Figure 8.** A). The HPLC of purified B1-L-6; B) ESI-MS spectrum of B1-L-6 calcd. for C_102_H_170_N_26_O_15_ 2000.65; found [M+2H]^2+^=1001.50; [M+3H]^3+^= 667.95.

**Supplementary Figure 9.** A). The HPLC of purified B1-L-7; B) ESI-MS spectrum of B1-L-7 calcd. for C_102_H_170_N_26_O_15_ 2000.65; found [M+2H]^2+^=1001.30; [M+3H]^3+^= 667.90.


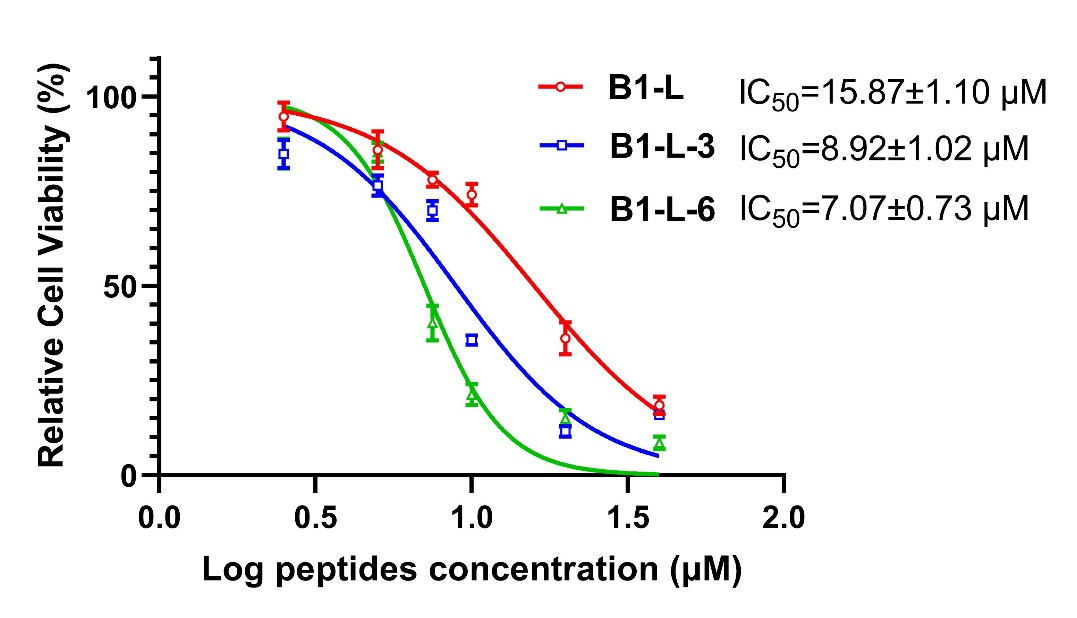


**Supplementary Figure 10.** Cytotoxicity in Nthy-ori 3-1 cell line of B1-L, B1-L-3 and B1-L-6. Data points were displayed as the mean value SEM of three repeated independent experiments.
